# Differentiation at the *MHCIIα* and *Cath2* Loci in Sympatric *Salvelinus alpinus* Resource Morphs in Lake Thingvallavatn

**DOI:** 10.1371/journal.pone.0069402

**Published:** 2013-07-24

**Authors:** Kalina H. Kapralova, Johannes Gudbrandsson, Sigrun Reynisdottir, Cristina B. Santos, Vanessa C. Baltanás, Valerie H. Maier, Sigurdur S. Snorrason, Arnar Palsson

**Affiliations:** Institute of Life and Environmental Sciences, University of Iceland, Reykjavik, Iceland; Temasek Life Sciences Laboratory, Singapore

## Abstract

Northern freshwater fish may be suitable for the genetic dissection of ecological traits because they invaded new habitats after the last ice age (∼10.000 years ago). Arctic charr (*Salvelinus alpinus*) colonizing streams and lakes in Iceland gave rise to multiple populations of small benthic morphotypes, often in sympatry with a pelagic morphotype. Earlier studies have revealed significant, but subtle, genetic differentiation between the three most common morphs in Lake Thingvallavatn. We conducted a population genetic screen on four immunological candidate genes *Cathelicidin 2* (*Cath2*), *Hepcidin* (*Hamp*), *Liver expressed antimicrobial peptide 2a* (*Leap-2a*), and *Major Histocompatibility Complex IIα* (*MHCIIα*) and a mitochondrial marker (D-loop) among the three most common Lake Thingvallavatn charr morphs. Significant differences in allele frequencies were found between morphs at the *Cath2* and *MHCIIα* loci. No such signal was detected in the D-loop nor in the other two immunological genes. In *Cath2* the small benthic morph deviated from the other two (*F_ST_* = 0.13), one of the substitutions detected constituting an amino acid replacement polymorphism in the antimicrobial peptide. A more striking difference was found in the *MHCIIα*. Two haplotypes were very common in the lake, and their frequency differed greatly between the morphotypes (from 22% to 93.5%, *F_ST_* = 0.67). We then expanded our study by surveying the variation in *Cath2* and *MHCIIα* in 9 Arctic charr populations from around Iceland. The populations varied greatly in terms of allele frequencies at *Cath2*, but the variation did not correlate with morphotype. At the *MHCIIα* locus, the variation was nearly identical to the variation in the two benthic morphs of Lake Thingvallavatn. The results are consistent with a scenario where parts of the immune systems have diverged substantially among Arctic charr populations in Iceland, after colonizing the island ∼10.000 years ago.

## Introduction

Processes of divergence and adaptation reflect evolutionary forces that alter the genetic make-up of populations over time [1]. While the bulk of these changes must be neutral, some are likely driven by natural selection. By identifying genes relating to adaptation we may be able to disentangle history, neutral forces and the contribution of positive and purifying selection on these evolutionary processes [2], [3]. One approach to identify such loci is to dissect the molecular genetics of major adaptations in highly divergent species [4], another is to compare genetic architecture of adaptive traits between closely related species or populations [5]. One of the advantages in studying recent (or ongoing) divergence is that relatively few genetic changes differentiate populations or sibling species, compared to the vast number of changes separating major taxa. A potential downside to this approach is that, on short evolutionary time scale, divergence is mainly shaped by drift and fine tuning of preexisting adaptations. However, certain study systems have the advantage of rapid evolution, for instance when species respond to geographic catastrophes or when they colonize novel habitats [6], [7].

Following the retreat of the last ice age cap (∼10,000 years ago) anadromous and freshwater fishes in the northern hemisphere invaded and explored new habitats [8]. In some cases streams and lakes provided novel niches, which the colonizing populations may have adapted to. Multiple species (white fish, three spine sticklebacks, several salmonids) show signs of repeated adaptive changes in independent waterbodies [9]–[13], some of which have been dissected genetically [14]–[17].

### Evolutionary Immunology of Fishes

The invasion into new habitats, changes from anadromous to “freshwater only” lifestyle, and sharing of habitat with other fishes provides novel challenges to the immune system of fishes [8]. The adaptive significance of immunological genes has been clearly illustrated. There are data supporting the role of frequency dependent selection, importance of local adaptation, the role of generalist vs. specialist lifestyle and parasites, involvement in assortative/disassortative mating and even magic trait sympatric speciation as defined by [18], see [19] for review.

Fish possess both an adaptive and an innate immune system. The Major Histocompatibility complex (MHC) are cell surface molecules (class I on most cells and class II on specialized cells) that are involved in pathogen recognition and are central to adaptive immunity [20]–[22]. The MHCII is a heterodimer protein made of an α and a β chain, each with two domains (α1 and α2, β1 and β2 respectively). MHC genes have been identified in many teleost species and in general the β chain tends to be highly polymorphic [23]. The favoured explanation is that the multitude of infectious agents and environmental heterogeneity favours heterozygotes and rare alleles, which through balancing or frequency dependent selection result in high MHC diversity [19]. MHC allele diversity can be reduced in fish populations, as a consequence of local adaptation [24], [25]. The distribution of *MHCIIα* alleles in Arctic charr is consistent with some degree of local adaptation [26], which will be studied further in this paper. Similarly data from brown trout (*Salmo trutta)* and Atlantic salmon (*S. salar*) how population differentiation in immunological genes, including TAP (Transporter associated with antigen processing) and interleukin-1 beta [27], [28]. Curiously MHCII genes have been lost in Atlantic cod and related species [29], whereas in the Salmonidae they were duplicated along with the whole genome about 25–100 millon years ago [30]. There are two MHCII regions in Salmonids (observed in Atlantic salmon and rainbow trout (*Oncorhynchus mykiss*)), and evidence suggests at least four *MHCIIα* copies can be expressed [31].

The innate immunity system constitutes an evolutionarily old defense strategy, as the majority of gene families involved in it are present throughout the animal kingdom [32]. Innate immunity depends on a wide array of recognition, signal transduction and defence molecules, which are thought to evolve fast in response to pathogens. For instance, a comparison of 12 Drosophila species genomes revealed signs of positive selection on protein sequence and gene copy number in the sensory and effector genes of the innate immunity [33]. Innate immunity is considered to be of key importance in combating infections in fish [21], [22]. Antimicrobial peptides (AMPs) play a major role in this system and in mammals these cationic peptides not only kill bacteria, but are multifunctional effectors of the innate immune system [34], [35]. Many AMPs have been identified in fish including Cathelicidins (Cath), liver expressed antimicrobial peptides (LEAP) and hepcidins (HAMP) [36]–[40]. In salmonids two types of Cathelicidins have been identified; Cathelicidin 1 and 2 [39]–[41]. Cathelicidins are generally encoded by four exons with the exception of *Cathelicidin 2* (*Cath2*) in the *Salvelinus* genus, which have lost exon 3. In fish Cathelicidins expression increases due to bacterial infection and the mature antimicrobial peptide has been shown to have bactericidal activity [39], [40], [42]–[44]. Several studies have shown signs of positive selection on AMPs (reviewed by Tennessen [45]), specifically on the charged amino-acids. Population genetic studies of the AMPs and other innate immunity genes are needed to elucidate the distinct selection pressures that shape these ancient defense systems.

### Arctic Charr Diversity and Resource Polymorphism

Arctic charr is a widespread circumpolar species. While it’s distribution reaches south along the coastal areas of the N-Atlantic it is best described as an Arctic species and indisputably the most cold tolerant of the salmonids [46]. In the high north Arctic charr is often found in very cold waters and lakes with limited productivity and with few or no other fish species present. A body of ecological studies document high diversity among Arctic charr populations (e.g. refs. in [46]–[48]), and many instances of resource polymorphism within lakes (see refs. in [8], [49], [50]). The favored explanation is that diversity arises via ecological specialization in habitat use and diet, facilitated by relaxed inter-specific competition, leading to morphological divergence among and within lakes [8], [51].

Icelandic Arctic charr descend from European charr [52] that colonized the island after the glacial retreat. Large parts of Iceland are constantly shaped by tectonic and volcanic activity which appear to have created special habitats for dwarf forms of Arctic charr that typically inhabit streams, ponds and lakes in the neo-volcanic zone that traverses Iceland from the south-west to the north-east. Kristjansson and coworkers have shown that in these habitats these small fish show similar phenotypes across locations, *e.g.* a typically benthic morphology, thus retaining a juvenile morphotype [53]. However, their evidence also shows that the morphological parallelism is incomplete [54], [55]. In lakes with two or more distinct morphs they usually conform to two types in terms of morphology (i.e. morphotypes), a pelagic and a benthic type, that typically reflect their modes of habitat utilization. Multiple lines of evidence show that these differences stem both from environmental and genetic causes [56]–[58].

The best studied and most extreme example of sympatric charr morps are the four morphs in Lake Thingvallavatn [59]. Two large morphs are found, a large benthivorous (LB-charr) and a piscivorous morph (PI-charr), and two small forms (morphs), a small benthivorous (SB-charr) and planktivorous morph (PL-charr). PL- and PI-charr, display a pelagic morphotype and are more inclined to operate in open water and feed on free swimming prey, planktonic crustaceans and small fish, respectively. The two benthic morphs show a benthic morphotype and mainly reside on the bottom, feeding exclusively on benthic invertebrates. The very small size of the SB-charr also allows them to utilize interstitial spaces and crevices in the littoral zone typically consisting of submerged lava which offers a rich source of benthic invertebrate prey. As would be expected from the clear cut ecological diversification of the morphs their macroparasitic fauna differs distinctively [60].

Population genetic studies based on variation in mtDNA revealed a common ancestry of Arctic charr in the Nordic countries, Ireland and Iceland [52]. Within Iceland, allozyme, mtDNA and microsatellite data reveal significant genetic differences between localities and in some cases between sympatric morphs, like the four morphs in Lake Thingvallavatn [61]–[63]. The genetic differentiation among the Thingvallavatn morphs is rather weak however, the average *F_ST_* over 10 microsatellites being 0.03, and a coalescence model suggests a scenario of early divergence with subsequent barriers to gene flow [63]. The strongest indication of genetic differentiation between sympatric charr morphs is a fixed difference in one microsatellite marker between two morphotypes in Lake Galtabol [64]. On a larger scale the available data suggest repeated evolution of dwarf forms (small fish with a benthic phenotype) in numerous Icelandic lakes and stream habitats in the neo-volcanic zone [53], [63].

Molecular genetics have also been used to address the developmental basis of morphotype differences in Icelandic Arctic charr [65], [66]. Macqueen and colleagues [66] conducted a study of the expression of 21 mTOR and growth regulation genes in 7 distinct Icelandic charr populations (thereof 5 with a small benthic morphotype), and revealed substantial divergence in gene expression of many pathway components. For instance mTOR is less and 4E-BP-1 more highly expressed in the populations of small benthic populations compared to other populations, a finding consistent with the role of these genes in protein synthesis and growth regulation [55], [66]. It is not clear whether those pathways are the foci of selection for changes in size and form, or realisitors of genes that promote dwarfism. Notably, considering our focus on immunological genes, the mTOR pathway is also involved in regulation of innate immunity [67], [68].

We hypothesized that local differences in habitat use and diet between the morphs in Lake Thingvallavatn and among other Arctic charr populations and morphotypes in Iceland could impact variation in important immunological genes. Using samples from all major phylo-geographic groups of Arctic charr [52] Conejeros and colleagues [26] reported on rich allelic variation at the *MHCIIα* locus within and between charr populations. Their data showed considerable shared diversity within populations and across a broad geographic range, but are also consistent with differentiation among populations reflected in unique haplotypes and frequency differences. Here we present a study on a smaller geographic scale analyzing variation in *MHCIIα* and four other innate immunity genes in Icelandic Arctic charr. Our focus was on the three most common sympatric morphs from Lake Thingvallvatn and 9 populations of small benthic, anadromous and lake resident charr from the neo-volcanic zone (south, west and north) in Iceland – that we studied previously with 9 microsatellites [63]. Thus in this study we could interrogate local differences in gene frequencies and probe geographic patterns in these loci in small benthic charr in Iceland. The results indicate marked differentiation between sympatric morphotypes in Lake Thingvallavatn in two loci, *Cath2* and *MHCIIα* that we investigated further. Our findings have bearing on the understanding of those unique sympatric Arctic charr morphotypes, and immune system diversity in organisms with evolutionarily recent resource polymorphism.

## Materials and Methods

### Sampling

**Figure 1:**
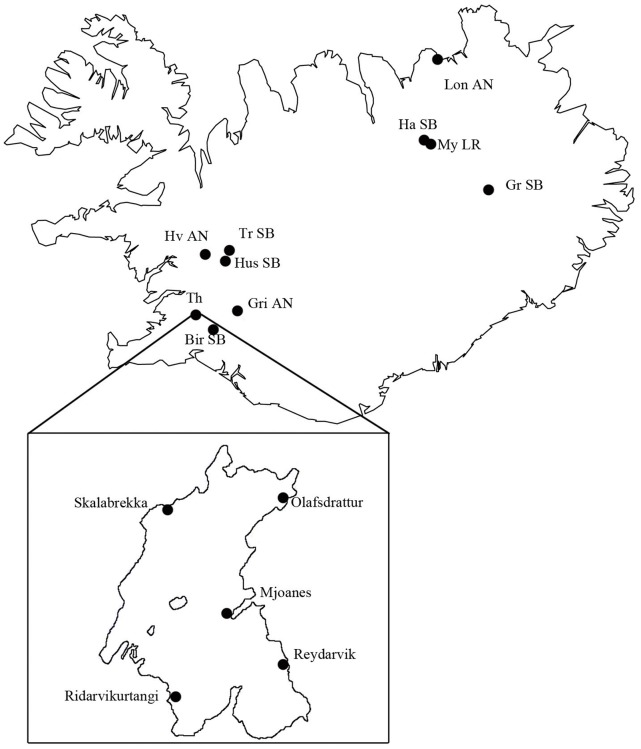
Sampling locations of Arctic charr in Lake Thingvallavatn and around Iceland. Fishes where collected in five locations within Lake Thingvallavatn (left), and from 9 other locations and populations around Iceland. In Lake Thingvallavatn, O: Olafsdrattur, M: Mjoanes, Re: Reydarvik, R: Ridvikurtangi and S: Skalabrekka. Around the island, either small benthic (SB) and lake resident (LR) or anadromous (AN) charr in Myvatn (My, LR), Haganes (Ha, SB), Lon (Lo, AN), Grafarlond (Gr, SB), Grimsnes (Gr, AN), Birkilundur (Bir, SB), Hvita (Hv, AN), Trussa (Tr, SB) and Husafell (Hus, SB).

Specimens came from three collections of Arctic charr from Icelandic lakes and rivers. First, we utilized a sample of 30 large bentivorous charr (LB-charr, not sexed) caught on their spawning grounds at Olafsdrattur, and a total of 406 spawning small benthivorous charr (SB-charr, 102 females/83 males) and plantkivorous charr (PL-charr, 83 females/115 males) caught at Olafsdrattur and four other spawning locations in Lake Thingvallavatn in October 2005 (Table 1, Figure 1, inset) (for details see Kapralova *et al.* [63] ). Second, we used another sample of 76 SB-charr (17 females/59 males), 102 PL-charr (51 females/males) and 17 LB-charr (1 female/16 males) collected in Olafsdrattur and Mjoanes, in September and October 2010 respectively. These two samples were pooled as our previous results [63] and the data from 2005, did not suggest genetic differentiation by location. The sampling in Lake Thingvallavatn focused on the SB and PL morphs, and the LB morph was mainly used for reference (hence the relatively lower sample size). For the 2010 sample, sex, fork length, weight, maturity and age were documented and parasite load (see below) assessed for every individual. DNA was extracted from a fin clip following a standard phenol-chloroform protocol. Third, we utilized samples from 9 populations of Arctic charr selected from a larger survey throughout Iceland collected in 2003–2006 (Table 1, Figure 1) previously described [63]. Those specimens were not sexed.

**Table 1:** Details on sampling locations and the number of individuals collected in 2005 and 2010.

Location	Morphotype	Code	Latitude	Longitude	2005	2010
Thingvallavatn	Large benthic	TH_LB	64°11	21°08	30	17
Thingvallavatn	Small benthic	TH_SB	64°11	21°08	185	76
Thingvallavatn	Planktivorous	TH_PL	64°11	21°08	198	102
Grimsnes	Anadromous	Gri_AN	64°00	20°53	27	
Birkilundur	Small benthic	Bir_SB	64°01	20°57	30	
Hvita	Anadromous	Hv_AN	64°42	20°59	35	
Trussa	Small benthic	Tr_SB	64°43	20°46	29	
Husafell	Small benthic	Hus_SB	64°41	20°52	31	
Lon	Anadromous	Lon_AN	66°05	16°55	27	
Grafarlond	Small benthic	Gr_SB	65°15	16°09	31	
Myvatn	Lake resident	My_LR	65°37	17°03	34	
Myvatn-Haganes	Small benthic	Hag_SB	65°37	17°03	35	

Fishing in Lake Thingvallavatn was with permissions obtained both from the owner of the land in Mjóanes and from the Thingvellir National Park commission. Ethics committee approval is not needed for regular or scientific fishing in Iceland (The Icelandic law on Animal protection, Law 15/1994, last updated with Law 157/2012). However, sampling was performed with University College Aquaculture Research Station (HUC-ARC) personnel. HUC-ARC has an operational license according to Icelandic law on aquaculture (Law 71/2008), that includes clauses of best practices for animal care and experiments.

### Molecular Work and Data Processing

We screened for sequence variation in four immunological genes: *Cath2, Leap-2a, Hamp* and *MHCIIα* among the three Thingvallavatn morphs (SB-, PL- and LB-charr). Moreover we studied a 510 bp region of the D-loop (starting at base 25 in the *S. alpinus* mtDNA reference genome, accession number NC_000861.1) as a putative neutral marker or marker of maternal lineage sorting. Loci were amplified by PCR with TEQ polymerase (Prokaria-Matis). We used previously published primers for *MHCIIα* [26] and new primers for *D-loop*, *Leap-2a*, *Hamp* and *Cath2* (Table S1), designed with Primer3 (http://primer3.wi.mit.edu/ [69]). The following PCR program was used for all primer pairs, except *MHCIIα.* Denaturation at 95°C for 5 min; 35 cycles of 95°C for 45 seconds; 45 seconds at a marker specific annealing temperature (Table S1); 1 min at 72°C, then a final step of 10 min at 72°C. For *MHCIIα* we used touchdown PCR, initial denaturation at 94°C for 5 min; 16 cycles of 94°C for 45 seconds, 62°C for 45 seconds (decreasing by 0.5°C every cycle), 1 min at 68°C; followed by 25 cycles of 94°C for 45 seconds, 53°C for 45 seconds, 1 min at 68°C; then a final step of 10 min at 68°C. PCR products were ExoSap purified, sequenced (BigDye) and run on an Applied Biosystems 3500xL Genetic Analyzer (Hitachi).

Raw sequencing data was base-called by Sequencing Analysis Software v5.4 with KBTMBasecaller v1.41 (Applied Biosystems), and run through Phred and Phrap [70], prior to trimming primer sequences, visual editing of ambiguous bases and putative polymorphisms in Consed [71]. Fasta files were exported and aligned with ClustalW (http://www.ebi.ac.uk/Tools/msa/clustalw2/, [72]) and manually inspected for alignment errors in Genedoc (www.psc.edu/biomed/genedoc) [73]. All sequences where deposited as Popsets in Genebank under the accession numbers KC590653-KC591103, KC591105-KC591218, KC591220-KC591303, KC591303-KC591626 and KC596075-KC596117.

### Genotyping *MHCIIα*

Due to potential duplications or deletions of *MHC* genes and the ancestral genome duplications in salmonids [30] the presence of *MHC* paralogous genes has to be investigated in charr. Initially we used the SAALDAA primers from Conejeros *et al.* [26], (Table S1) that pick up part of exon 2 and intron 2 of *MHCIIα*, but obtained several satellite bands. To confirm the amplification of MHC, bands of various sizes (from a non-optimized PCR) were cloned into a TOPO vector (Invitrogen) and sequenced. Blastn was used to find related sequences in Genebank (NCBI – nucleotide collection – at latest in April 2013). We obtained bands from 4 size ranges. Most importantly, a ∼400 bp fragment sequenced from 2 individuals (10 clones from each) yielded 3 different fragments of *MHCIIα* (Table S2). One of these fragments, represented by 5 clones from each individual, was 99% identical to Saal-DAA*0801 [26]. The other two versions, each restricted to one individual, had 98% and 99% identity to Saal-DAA*0305/0306/0307 and Saal-DAA*0305 [26], respectively (Table S2). The largest band (∼720bp) was only present in ∼1% of the samples and all ten clones from this band were identical to *MHCIIα* haplotype Saal-DAA*0104 (intron haplotype hap1 as defined by [26]. Two smaller fragments, ∼250 bp and ∼150 bp, contained mixed products of various origins unrelated to *MHCII*.

The PCR protocol was optimized to reduce unspecific small auxiliary bands (see above) and we proceeded with PCR and direct sequencing. The first 32 *MHCIIα* sequences from Lake Thingvallavatn (2005 sample) were amplified with the SAALDAA primers, and sequenced with both forward and reverse primers (error rate of Single nucleotide polymorphisms (SNP’s) called was <0.1%). Subsequently only the forward primer was used to sequence the PCR products. In total PCR and direct sequencing of 413 individuals from the 2005 sample gave sequences of three major types. Those corresponded to the large fragment (intron haplotype hap1) and the two versions (similar to Saal-DAA*0303 and Saal-DAA*0305), that we denote as second intron haplotypes 14 and 15. The fragment identical to Saal-DAA*0801 was never observed. PCR and direct sequencing clearly revealed individuals heterozygotic for a single base insertion/deletion polymorphism (indel) in the intron. To us the data suggest that two *MHCIIα* paralogous genes are present in Arctic charr, with hap14, hap15 and possibly hap1 being alleles of one paralog. The optimized PCR preferentially amplifies this paralog. This is supported by two observations. First, in the direct sequencing we never observe Saal-DAA*0801 (the two suspected paralogs are easy to distinguish) and second, the indel in the second intron conforms to Hardy Weinberg Equilibrium, within each morph (see below).

Because of low DNA availability and degradation in the 2005 Icelandic lake samples, we designed new primers (Table S1.) that gave a shorter amplicon and none of the satellite bands. With those primers fragments of *MHCIIα* from 6 individuals were amplified, cloned and sequenced (as before). We sequenced on average 8 clones per individual and in all cases the genotyping was in perfect concordance with the genotyping from PCR and direct sequencing. The suspected paralogous copy of *MHCIIα* (similar to Saal-DAA*0801) was found in a low proportion of the clones (5/45 sequences). The 2010 sample from Lake Thingvallavatn and the 9 Iceland wide populations were amplified and sequenced with these primers. Although there is a potential for ascertainment bias, as samples from two years (2005 and 2010) were genotyped with different primers, the results do not indicate a bias; the frequency of the indel variation was not statistically different between years (tested within morphs, see details below). Finally, we also did a restriction enzyme analysis, that could distinguish hap14 and hap15 on basis of a G/A polymorphism 13 bp down stream of the indel (TGAATGAATCAATAGGATTAATGTAGTAAA(A/−)TAGTCACCTCACT(G/A)TAACCTCTCACATGTTGTATCATCTGTGGTATGG). These two polymorphisms were fully coupled in the sequencing data. This restriction digest of 28 individuals (equal number from 2005 and 2010) was in perfect concordance with the PCR and sequencing data.

### Population Genetic Analyses

Tassel version 2.0.1 (www.maizegenetics.org) [74] and DNAsp 4 (www.ub.edu/dnasp/) [75] were used to calculate and analyze population genetic statistics. Tests of Hardy Weinberg proportions, allele and genotype frequencies between morphs, locations and were implemented in R (version 2.12, R Development Core Team, 2011). Arlequin v3.5.1.2 was also used to estimate *F_ST_* [76]–[78]. We tested determinants of genetic differentiation between morphs within Lake Thingvallavatn with analyses of molecular variance (AMOVA) using Arlequin. We analyzed variation in 3 amplicons (*D-loop*, *Cath2* and *MHCIIα*), within Lake Thingvallavatn with a two level AMOVA with morph (LB, SB, SP) as a categorical variable, split by sex or sampling location.

The genetic relationships between and within morphs were estimated with an unrooted neighbor-joining tree. The tree was constructed using Cavalli–Sforza’s genetic distances obtained from nine microsatellite loci [63] with the program NEIGHBOUR available in PHYLIP3.69 [79]. Confidence intervals were estimated by 1000 bootstrap replicates.

### Parasite Analyses

The 2010 samples from Lake Thingvallavatn were used to assess infection rates and loads of the eye parasite *Diplostomum sp*., the intestine parasite *Eubothrium salvelini*, Nematodes and *Diphyllobothrium sp*. Both eyes were extracted from each individual. The contents of each eye was poured on a flat slide, covered with a slip and processed under a Leica KL200 LED microscope at 2X magnification. The slide field was divided into 45 blocks, and the average number of metacercaria of *Diplostomum sp*. was estimated. We first screened all blocks, and in case of even distribution among them, counted the metacercaria in 5 randomly selected blocks, and then calculated average infection rate. In case of non-uniform distribution or low infection we counted the parasites in all 45 blocks. We recorded both counts and used an infection scale [60] ; 0 =  total absence of parasites; 1 = 1 or fewer parasites per blocks; 2 = 1 to 3 individuals per block; 3 = 4 to 10 parasites per block and 4 represented more than 10 *Diplostomum sp*. individuals per block. The estimation was done by a single observer (S. Reynisdottir) on a single eye per specimen. The correlation of infection rate between eyes was high (Pearson *r* = 0.75, p<0.005, for 25 pairs of eyes studied).

Infections by *Eubothrium salvelini* were assessed by carefully extracting the liver, stomach and intestine and documenting the presence or absence of the adult tapeworm. Infections of nematodes and plerocercoids of *Diphyllobothrium sp*. were estimated by counting individual nematodes and *Diphyllobothrium* cysts internal cavities and linings of flesh [60], [80]. The *Diphyllobothrium sp.* infection rate was scored using the following infection scale: 0 =  the total absence of parasites; 1 = 1 to 3 per individual; 2 = 4 to 7 per individual and 3 equaled more than 8 parasites per individual. For Nematodes the number per individual was recorded. All data on intestinal parasites were obtained by a single observer (C. B. Santos). Data of the 2010 and 2005 samples from Lake Thingvallavatn were deposited in the Dryad Repository: http://dx.doi.org/10.5061/dryad.81884.

### Statistical Analyses of Parasite Infections

Statistical analyses were performed in R. The effects of morph, sex and weight on the load of individual parasite species was investigated with multivariate regression. Summary statistics were calculated for weight, age and parasite loads separately for each morph. Sex ratio was also calculated. For *Diphyllobothrium sp*. and *Diplostomum sp.* mean relative density (MRD) was calculated [60]. Statistical models for parasite load were applied to morph pairs to test for difference between the morphs. As parasite loads turned out to be different between morphs tests for other factors affecting the load were applied to the morphs separately. The models had the general structure:
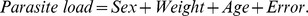



A term for genotype was also added to evaluate the impact of *MHCIIα* variation within morphotypes. The ANOVA function from the *car* package [81] was used to perform F-tests and log-likelihood tests. Raw counts of *Diphyllobothrium sp*. and *Diplostomum sp*. were analyzed by multivariate linear regression and variable effects tested with an F-test. The infections were also summarized with an infection scale [60] and analyzed using multinomial logit regression fitted with neural networks [82], with consistent results. Effects were tested with log-likelihood tests. Logistic regressions were applied to Nematodes and *Eubothrium salvelini* occurrence and effects were tested with log-likelihood test.

## Results

### Nucleotide Polymorphism in Arctic Charr Morphs in Lake Thingvallavatn

Different molecular markers have revealed significant but weak genetic differentiation among the Lake Thingvallvatn charr morphs [61]–[63]. Here we make use of genetic material from individuals previously typed for 9 microsatellite markers [63] to explore variation in four immunological loci, and test for indications of population differentiation.

Four segregating sites were observed in the mitochondrial *D-*loop, but nucleotide diversity was rather low (Table 2). Of the four substitutions only one (m38A>G) had significant difference in frequency between PL and SB (*χ^2^* [*_1_*] = 9.36, p = 0.002). The *F_ST = _*0.001, which was lower than the *F_ST_* for microsatellites between charr morphs in Lake Thingvallavatn [63]. A comparison with *S. alpinus D*-loop in genebank [52], [83] shows that none of the four *D*-loop sites are restricted to Iceland. Analyzes of molecular variance (AMOVA) confirm that the observed variation in this part of the mtDNA of Lake Thingvallavatn charr is not affected by morph, sex or sampling location (Table 3).

**Table 2:** Polymorphism in the mitochondrial D-loop and three immunological genes.

Gene/region	Morph	Size (bp)	N	S	Indel	*π*	*θ*	Haplotypes
D-loop	All	509	406	4	0	0.001	0.001	7
	PL	509	190	3	0	0.001	0.001	4
	SB	509	216	4	0	0.001	0.001	7
*Hamp* 5′ UTR	PL/SB*	454	12	0	0	0.000	0.000	1
*Leap-2a* 3′ UTR	All	559	15	4	1	0.001	0.004	3
	PL	559	8	2	0	0.001	0.003	2
	SB	559	7	3	1	0.002	0.003	3
*Cath2* (intron 2)	PL/LB/SB*	219	258	0	0	0.000	0.000	1
*Cath2* (peptide)	All	396	258	3	0	0.001	0.001	4
	PL	396	138	2	0	0.000	0.001	3
	LB	396	35	1	0	0.000	0.001	2
	SB	396	86	3	0	0.001	0.001	4
*Cath2* (3′ UTR)	All	407	17	2	1	0.002	0.002	3
	PL	407	6	1	0	0.002	0.001	2
	SB	407	11	2	1	0.002	0.002	3

S: Segregating sites. Indel: Segregating insertion/deletion polymorphism. *π:* The average number of nucleotide differences per site. *θ:* Wattersons estimator of diversity per site. *The data from different morphs are summarized together as no differences in frequence were observed.

**Table 3:** Analyses of molecular variance (AMOVA) of three loci by morphotypes (PL, LB and SB collected in 2005) and either location or sex.

Gene	Terms	d.f.	Sum of squares	Variance	Variation (%)	Fixation index	p-value
D-loop*	Among morphs	1	0.3	0	0.83	FSC : −0.01	ns.
	Among locations within morphs	7	0.39	0	−1.44	FST : −0.01	ns.
	Within locations	389	58.58	0.15	100.62	FCT : 0.01	****
	Total	397	59.27	0.15			
	Among morphs	1	0.17	0	0.4	FSC : −0.01	ns.
	Among sexes within morphs	2	0.11	0	−0.66	FST : 0	ns.
	Within sexes	393	58.17	0.15	100.26	FCT : 0	****
	Total	396	58.45	0.15			
*Cath2*	Among morphs	2	4.56	0.02	12.64	FSC : 0.03	****
	Among locations within morphs	7	2.2	0.01	3.02	FST : 0.16	**
	Within locations	253	42.18	0.17	84.34	FCT : 0.13	*
	Total	262	48.94	0.2			
	Among morphs	2	4.56	0.03	13.48	FSC : 0.01	****
	Among sexes within morphs	2	0.45	0	0.47	FST : 0.14	ns.
	Within sexes	258	43.94	0.17	86.05	FCT : 0.13	****
	Total	262	48.94	0.2			
*MHCIIα*	Among morphs	2	50.76	0.22	63.2	FSC : 0.03	****
	Among locations within morphs	8	0.88	0	−0.13	FST : 0.63	ns.
	Within locations	402	50.93	0.13	36.92	FCT : 0.63	***
	Total	417	102.57	0.34			
	Among morphs	2	51.44	0.22	64.06	FSC : −0.01	****
	Among sexes within morphs	2	0.04	0	−0.33	FST : 0.64	ns.
	Within sexes	408	50.91	0.12	36.28	FCT : 0.64	****
	Total	412	102.39	0.34			

* Only PL and SB were sequenced for the D-loop. d.f.: Degrees of freedom. Significance: ns. p>0.05,

* p<0.05,

** p<0.01,

*** p<0.001,

**** p<0.001.

We screened three innate immunity genes *Hamp*, *Leap-2a* and *Cath2* for nucleotide variation. The 454 bp *Hamp* amplicon, positioned in the untranslated 5′-region, proved invariant in a set of 12 specimens (6 PL- and 6 SB-charr). Four segregating sites and one insertion/deletion polymorphism (indel) were found in the 3′UTR of *Leap-2a*. These were at approximately equal frequency in SB- and PL-charr. The *Hamp* and *Leap-2a* genes where not studied further. Of the three regions surveyed in the *Cathelicidin* gene (spanning ∼1 kb), only the peptide region showed frequency differences between morphs (Table 2) urging further investigation. The sequenced part of intron 2 was invariant in the sample, whereas the three mutations (one indel and two SNPs) in the 3′UTR were at about the same frequency in both morphs.

**Figure 2:**
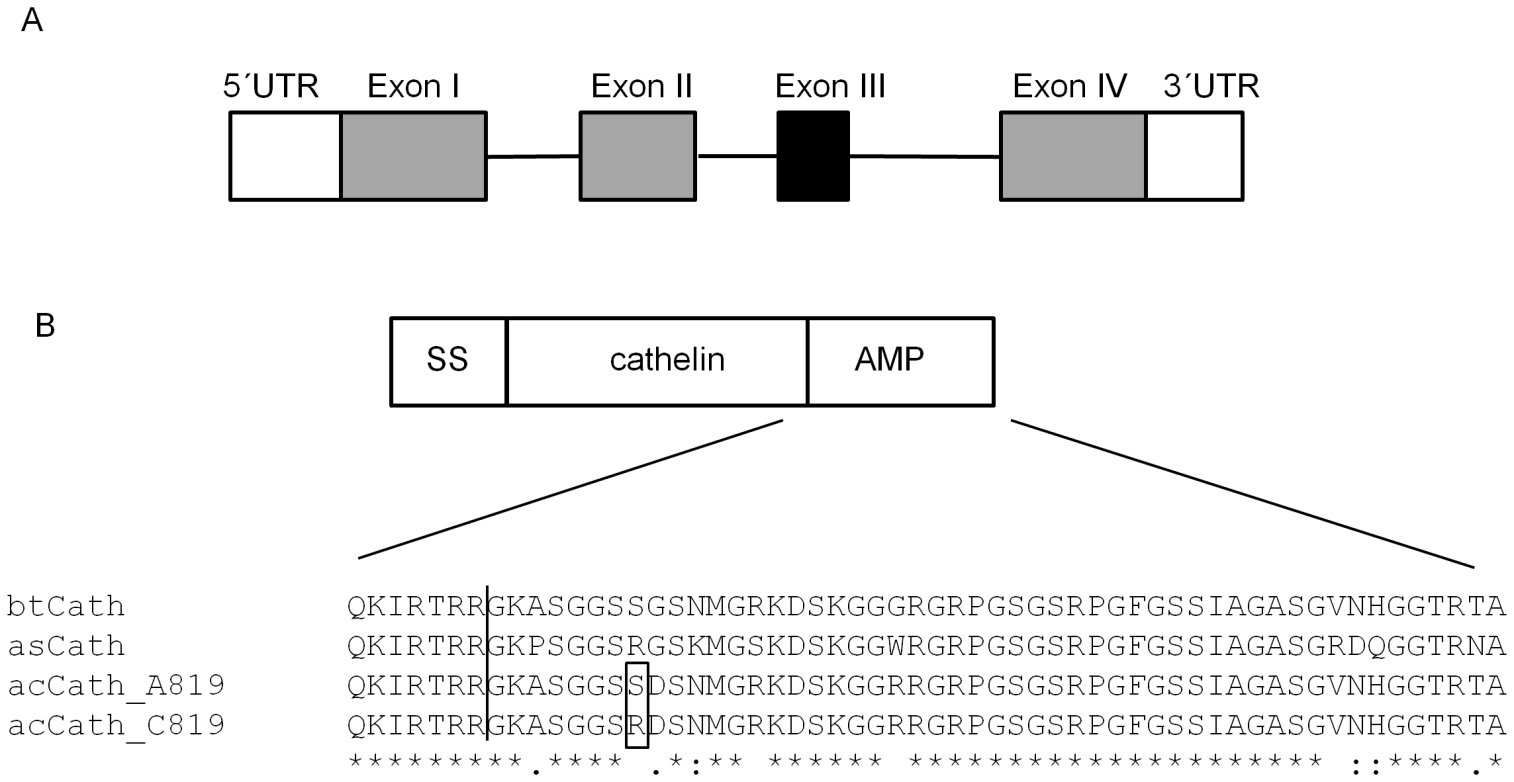
Polymorphism in the antimicrobial peptide Cathelicidin 2. *Cathelicidins* have a conserved 4 exon structure (A) with the exception of the *Salvelinus* Cathelicidins type 2 which have lost exon 3 (marked black). The peptides (B) are produced as pre-pro-peptides, where exon 1–3 encode the signal sequence (SS) and the conserved Cathelin region, while exon 4 encodes the processing site and the mature antimicrobial peptide (AMP). An amino acid alignment of this region for Cathelicidin 2 of Atlantic salmon (asCath), brook trout (btCath) and Arctic charr (acCath) shows the predicted processing site (vertical line) and the observed polymorphism (predicted peptide position 115) in Icelandic Arctic charr. Identical amino acids are marked with *, amino acids with a similar and somewhat similar function are marked with : and. respectively.

Sequencing of the antimicrobial peptide encoding region of *Cath2* in 264 individuals from Lake Thingvallavatn 2005 revealed three variant sites (including one singleton). One mutation (g558C>A) was found in intron 2. Another (g819C>A) was found in the exon encoding the mature antimicrobial peptide (in cathelicidins this region is on exon 4, but due to the lack of exon 3 in charr *Cath2* [40], it is encoded by the third exon in *S. alpinus,* Figure 2A). This mutation is predicted to lead to an amino acid replacement in the mature peptide (replacement of arginine by serine at position 115, Figure 2B). This alters the charge of the peptide, from +8 to +7.

We compared the frequency of the two mutations among morphs, sex and sampling locations in Lake Thingvallavatn. The g558C>A is largely restricted to the SB morph (11.3% frequency); it is not found in the LB-charr and only present in two of 134 PL-charr. The more common g819C>A variant shows significant frequency differences between morphs (*χ^2^* [*_2_*] = 43.91, p<0.0001). The A allele is at 27% frequency in SB-charr, but is rarer in LB- (5.7%) and PL-charr (6.4%). This translates into an *F_ST_* of 0.17 (p<0.0001) between the SB- and PL morphs, and *F_ST_* = 0.13 (p<0.0001) between the LB and SB samples. No differences in allele frequency where found between PL- and LB-charr, sexes or sampling locations. Analyses of Molecular Variance (AMOVA) confirmed these patterns (Table 3).

### *MHCIIα* Variation in Lake Thingvallavatn

Due to the structural richness of MHC regions and the fact that the common ancestor of salmonids underwent a whole genome duplication, studies of MHC variation in those species are rather complicated. We tackled this by genotyping with PCR and direct sequencing, and assessed the specificity and reproducibility of this genotyping method by cloning and restriction enzyme assays.

We concentrated on the highly variable intron 2 of *MHCIIα* [26], by DNA sequencing of 413 charr (LB, SB and PL) from Lake Thingvallavatn. There was high degree of polymorphism, with many segregating mutations (10 SNPs and 2 indels in ∼300 bp). Two major and two minor versions of *MHCIIα* were identified. The two major haplotypes hap 14 and hap 15 are quite distinct, being separated by 6 segregating sites and 1 indel. These polymorphism were were described by Conejeros *et al.* [26], but the haplotypes involving them are unique and probably arose by recombination. In addition two rare versions were observed, hap16 (just one site diverged from hap14) and hap1 (Saal-DAA*0104) which contains a Hpa retrotransposon [26]. The hap1 and hap16 haplotype were extremely rare in all morphs, for instance hap1 was found in four SB-charr from 3 sampling locations (1.08%) and one LB-charr (1.67%). Our analyses focused on the two dominant haplotypes, hap14 and hap15.

As described in Materials and Methods, the cloning results suggest the presence of two distinct *MHCIIα* paralogs in Arctic charr in Iceland. One of these was never observed with the PCR and direct sequencing, but only detected in the cloning (prior to PCR optimization). The hap14 and hap15 haplotypes are readily distinguishable based on several markers, such as the indel in the intron. We are quite certain that these are allelic variations (true haplotypes, not paralogous genes) because Hardy Weinberg proportions are respected for the indel polymorphism in *MHCIIα* intron in all three morphs in Lake Thingvallavatn (LB: *χ^2^* [*_1_*] = 0, p = 1, SB: *χ^2^* [_2_] = 1.77, p = 0.4, PL: *χ^2^* [_2_] = 6.2, p = 0.05). Furthermore restriction enzyme analysis of 28 individuals was in perfect concordance with the PCR and sequencing data.

As predicted [19], [27] the nucleotide diversity was higher in *MHCIIα* than in the other sequences studied; π was an order of magnitude higher than for *Cath2* and the D-loop (Table 2 and below). We found large differences in *MHCIIα* frequencies among the three morphs studied from Lake Thingvallavatn (Table 4), with hap15 being dominant in both benthic morphs, 93.5% and 88.3% in SB- and LB-charr respectively. In contrast hap15 was at 22% frequency in the pelagic morph (PL). This translates into an *F_ST_* of 0.56 (p<0.0001) between PL- and LB-charr, 0.67 between PL- and SB-charr (p<0.0001), and unsignificant *F_ST_* between the two benthic morphs. This represents the strongest genetic differentiation reported to date between any of these three sympatric morphs. These findings were further supported by AMOVA, the effect of morphotype (benthic versus pelagic) dominating the explained variance (above 60%), while sex and sampling location did not have significant effects (Table 3).

**Table 4:** The frequency of the three most common *MHCIIα* haplotypes in the arctic charr morphotypes from Lake Thingvallavatn sampled in 2005 and 2010.

	LB		SB		PL	
Haplotypes	2005	2010	2005	2010	2005	2010
hap1	1 (1.7%)	0 (0.0%)	4 (1.1%)	0 (0.0%)	0 (0.0%)	0 (0.0%)
hap14	6 (10.0%)	5 (14.7%)	20 (5.4%)	2 (1.3%)	309 (78.0%)	155 (76.0%)
hap15	53(88.3%)	29 (85.3%)	346 (93.5%)	150 (98.7%)	87 (22.0%)	49 (24.0%)
N	30	17	185	76	198	102

N: the total number of fishes genotyped in each sample.

**Figure 3:**
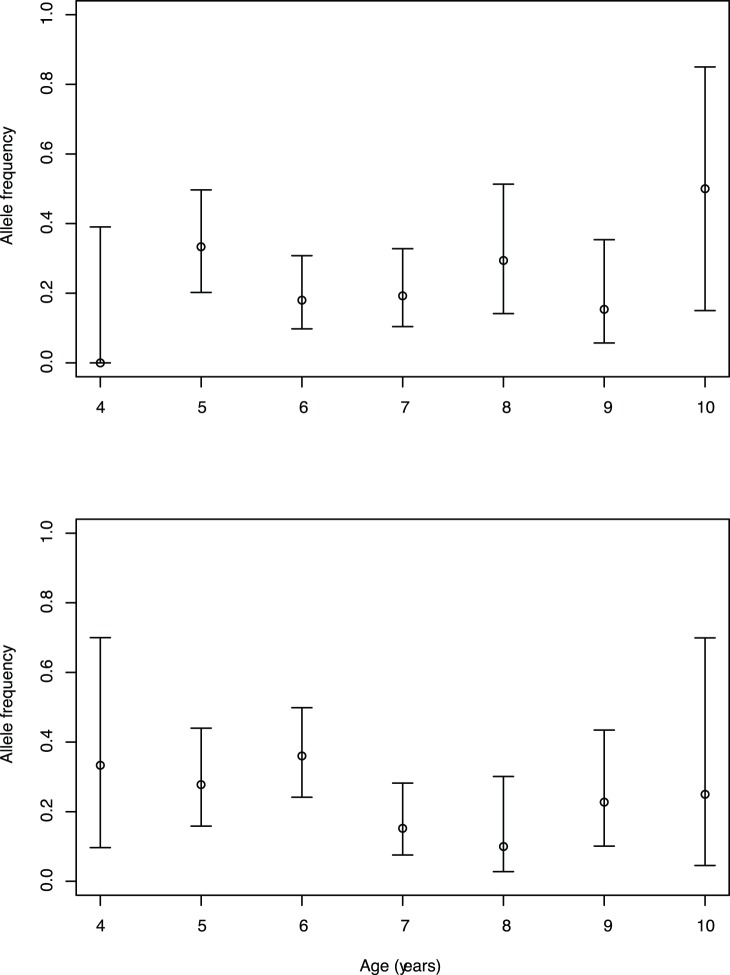
Frequency of *MHCIIα.*variations in PL-charr from 2005 and 2010 by age classes. The frequency of *MHCIIα* hap14 (with 95% confidence intervals) by age of PL charr, collected in years 2005 (A) and 2010 (B) at the spawning grounds in Lake Thingvallavatn.

This strong difference in *MHCIIα* frequency between morphotypes prompted several questions. Is the frequency difference consistent between years? What is the geographic distribution of variation in *MHCIIα* within Iceland? Do the haplotypes correlate with phenotypic attributes? We set out to answer these questions. Some hypotheses of MHC evolution involve temporal dynamics, for instance due to frequency dependent selection [19]. To evaluate this we used two approaches. We first compared the frequency of *MHCIIα* hap14 in the three morphs (PL-, LB- and SB-charr) in two cohorts sampled in 2005 and 2010 (Table 4). On all three morphs the haplotype frequencies were similar for the two years, *χ^2^*[*_1_*] = 0.0301, p = 0.9, *χ^2^* [*_1_*] = 0.08, p = 0.8, *χ^2^*[*_1_*] = 3.65, p = 0.06, for PL-, LB- and SB-charr, respectively. The age distribution was similar in the fishes collected, for instance the average age in PL sampled in 2005 and 2010 was 6.94 and 6.96 years respectively (weighted t-test, p = 0.96). No significant differences in haplotype frequency between years (*χ^2^* [*_2_*] = 0.59, p = 0.74) or age-classes were observed within morphs (Figure 3) (*χ^2^* [*_12_*] = 17.4, p = 0.13 for 2005 and *χ^2^* [*_12_*] = 10.5, p = 0.57 for 2010).

### *Cath2* and *MHCIIα* Polymorphism Across Morphotypes and Geographic Regions

**Figure 4:**
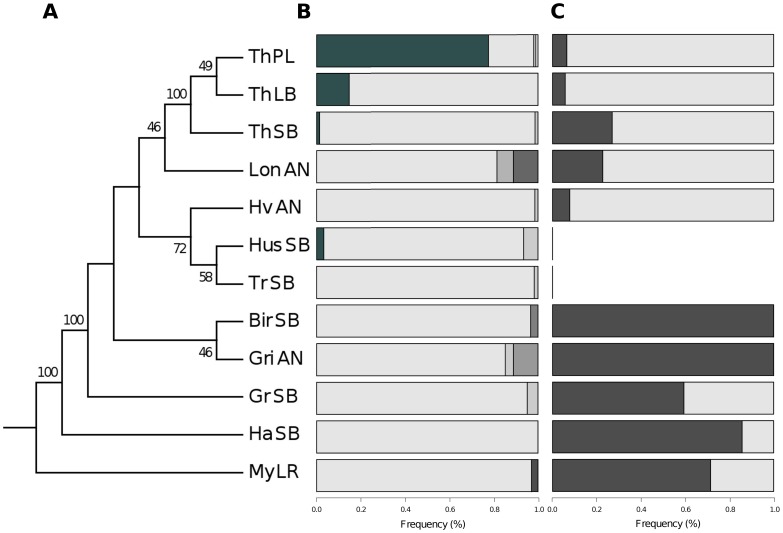
Arctic charr population history and variation in *Cath2* and *MHCIIα*. A) A genealogy of the sampled populations was built from 9 microsatellite markers and the confidence intervals were estimated by 1000 bootstrap replicates [63]. B) Frequencies of the *MHCIIα* intron haplotypes (hap 14 is dark, hap15 light gray, rare haplotypes are in intermediate shades of gray). C) The frequency of *Cath2* g819A (dark). Due to limited DNA available, the marker could not be typed in Husafell and Trussa. The same individuals where genotyped for all markers.

As the frequency of variants both in *Cath2* and *MHCIIα* deviated significantly between morphs within Lake Thingvallavatn, we wanted to know if the observations reflect a local or a broader geographic or ecological pattern. Our previous microsatellite study [63] enabled inference of relatedness among 9 Arctic charr populations from the north, west and south of Iceland (Figure 1 and 4A). We surveyed variations in both genes in those small benthic, anadromous and lake resident populations and superimposed on the microsatellite based tree.

There was very little polymorphism in *MHCIIα* in other populations and lakes, at maximum 3 haplotypes in each population (Table 5). The hap14 haplotype which dominated in the PL in Lake Thingvallavatn was only found in one other population (SB from Husafell), at 3% in 2 individuals (Figure 4B). The other haplotype (hap15), most common in the LB and SB morphs in Lake Thingvallavatn, dominated all other populations (average frequency 94%, lowest 81%). Several other haplotypes were observed, but all are one or few bases removed from hap15 and at very low frequency. The results show clearly reduced variation in this locus in Icelandic stocks of Arctic charr, except in the sympatric morphs in Lake Thingvallavatn. Summaries of nucleotide diversity reveal this pattern, as *π* (which responds to frequency and diversity of haplotypes) is larger in PL-charr from Lake Thingvallavatn than in the other charr populations surveyed (Table 5).

**Table 5:** Nucleotide diversity in *MHCIIα* in Lake Thingvallavatn 2010 sample and 9 other populations around Iceland.

Location	Size (bp)	S	*π*	*θ*	*Haplotypes*
TH_LB	293	8	0.013	0.014	3
TH_SB	293	8	0.003	0.010	3
TH_PL	293	10	0.018	0.014	4
All LakeThingvallavatn	293	10	0.024	0.012	4
Gri_AN	293	7	0.008	0.011	3
Bir_SB	293	2	0.001	0.003	2
Hv_AN	293	3	0.002	0.004	2
Tr_SB	293	3	0.002	0.005	2
Hus_SB	293	8	0.009	0.013	3
Lon_AN	293	3	0.003	0.005	3
Gr_SB	293	3	0.005	0.005	2
My_LR	293	2	0.001	0.003	2
Hag_SB	293	0	0.000	0.000	1
All Iceland w/oLake Thingvallavatn	293	12	0.003	0.011	8

S: the total number of segregating sites. *π*: The average number of nucleotide differences per site. *θ*: Wattersons estimator of diversity per site. See Table 1 for population identification code.

The *Cath2* g819C>A was genotyped in 7 populations (105 individuals total) and its frequency differed significantly between them (*χ^2^* [*_6_*] = 91.92, p<0.0001, Figure 4C). The g819C>A was dominant and even fixed in several small benthic charr populations (Birkilundur 100%, Haganes 86% and Grafarlond 59%). Recall, within Lake Thingvallavatn the variant was at highest frequency in the SB morph (27%), but lower in the other two. However g819C>A was also fixed in the anadromous Grimsnes population in the south of Iceland, and at high frequency in the lake resident population of large charr in Myvatn (71%) in the north. This translates into high interlocal *F_ST_*, for instance 0.85 between the anadromous populations in Hvita and Grimsnes. The average *F_ST_* for *Cath2* among all the populations was 0.29, while the average *F_ST_* for microsatellites was 0.245 [63]. While the frequencies of the *Cath2* g819A certainly differ between the populations, the *Cath2* locus is not associated with morphotype, as for instance g819A is fixed in both anadromous and small benthic populations. However, the *Cath2* variation may correspond, to some extent, to the relatedness of populations (Figure 4). Note however that not all branches in the tree have strong bootstrap support. Finally, there is no concordance between the variation in the two loci (*MHCIIα* and *Cath2*), and no linkage disequilibrium was observed between *Cath2* and *MHCIIα* variations and the microsatellites (*χ^2^* [*_2_*] = 0.11, p = 0.94).

### Tests of Association between *MHCIIα* Variation and Macroscopic Parasitic Infections

Frandsen and colleagues [60] reported a difference in parasite infection rate and prevalence between the four morphs in Lake Thingvallavatn. Can the differences in *MHCIIα* allele frequencies between the PL morph and the benthic morphs in Lake Thingvallvatn be driven by habitat-specific selection, caused by marked differences of infectious agents in habitat and diet? In immunity MHCII presents antigens of pathogens such as parasites [20], which may lead to evolutionary change [19]. We tested whether the *MHCIIα* variation is related to infection rate/prevelance of four classes of macroscopic parasites (*Diphyllobothrium sp., Diplostomum sp.,* parasitic nematodes and *Eubothrium salvelini*), in Lake Thingvallavatn charr. We sampled PL- (102), SB- (76) and LB charr (17) in the fall of 2010, screened for parasites and ascertained *MHCIIα* haplotypes. The pattern of parasite infection rate and prevalence (Table 6) is consistent with previous reports [60], with the *Diplostomum sp.* being most common in LB- and SB-charr, but the other three parasites infecting a very high fraction of PL-charr. This was confirmed by a generalized linear models analyses (Table 7), which also revealed the effects of age (*Eubothrium salvelini* in PL charr, *Diplostomum sp.* in SB- and LB charr), weight (*Diplostomum sp.* in SB- and LB charr and *Diphyllobothrium sp.* in PL charr) and sex (only significant for Nematodes in PL charr). We added a term for the genotype, to test the effects of *MHCIIα* on each of those parasite types. This was only done for the PL morph as there was almost no segregating variation in the benthic morphs. The genotype terms were not significant, neither as a class or quantitative variable (Table 7). The models were evaluated both on a parasite-scoring-scale and raw counts, with consistent results (Table 7). For exploration we also tested interaction of genotype with other terms, which yield borderline significance for Genotype by Sex interaction with nematodes (p = 0.07). Considering the number of tests preformed and the poor replicability of genetic interaction terms [84] this is almost certainly a spurious association. In summary, the data do not suggest that infection rate (or infection intensity) of those four parasite classes is affected by the frequency of *MHCIIα* alleles in Lake Thingvallavatn charr.

**Table 6:** Parasite infection rate in Lake Thingvallavatn Arctic charr in 2010.

		Morph		
Parasite	Measure	SB	LB	PL
*Diphyllobothrium sp.*	MRD	0.07	0.02	1.25
	Prevalence	15/113	5/19	125/131
	Count	0.22	0.4	10.13
	Score	0.15	0.37	2.32
*Diplostomum sp.*	MRD	46.84	8.69	9.93
	Prevalence	109/113	19/19	131/131
	Count	192.1	178.3	70.3
	Score	2.10	2.26	1.53
Nematodes	Prevalence	1/82	1/15	55/105
*Eubothrium salvelini*	Prevalence	6/82	2/15	68/105

MRD: mean relative density. Both count and score are summarized by arithmetic means.

**Table 7:** Generalized linear model analyses of the contribution of morph, sex, weight, age and *MHCIIα* genotype on parasite infections in Lake Thingvallavatn charr in 2010.

Parasite	N	Morph	Weight	Age	Sex	*MHCIIα*
*Diphyllobothrium sp.*	263	PL vs. SB***; LB vs. PL***	PL**	ns.	ns.	ns.
*Diplostomum sp.*	263	PL vs. SB***; LB vs. SB***	SB***; LB***	SB*; LB***	ns.	ns.
Nematodes	202	PL vs. SB***; LB vs. PL**	ns.	ns.	PL*	ns.
*Eubothrium salvelini*	202	PL vs. SB***; LB. vs. PL***	ns.	PL*	ns.	ns.

Significance: ns. p>0.05,

* p<0.05,

** p<0.01,

*** p<0.001.

## Discussion

The sharp distinction in form, size and ecology between the four sympatric Arctic charr morphs in Lake Thingvallavatn [8], [59] calls for explanation. Earlier studies found evidence of subtle but significant genetic differentiation among the morphs within the lake [50], [61]–[63], [85]. Here we report substantial genetic differentiation among the morphs within the lake, in two of the four immunological genes investigated (*Cath2* and *MHCIIα*). The pattern of divergence is not the same for both loci. In *Cath2* the strongest differentiation is between SB charr and the other two morphs studied (LB- and PL charr). Whereas in the case of *MHCIIα* the PL charr deviates markedly from the two benthic morphs within the lake, which have very similar haplotype frequencies. No differentiation was detected in two other innate immunity genes (*Hamp* and *Leap-2a*) nor the D-loop. The lack of association between mtDNA haplotypes and morphotypes, is consistent with results on variation in Arctic charr (dwarf and large forms) in 56 Siberian lakes [83]. Allele frequency differences can be caused by neutral and selective forces, but several studies have documented the impact of selection on immunological genes, with most focus on MHC loci [19], [33], [45].

### Which Evolutionary Forces Shaped the *MHCIIα* and *Cath2* Variation in Iceland?

We observe large frequency differences of the *MHCIIα* haplotypes in the three sympatric morphs in Lake Thingvallavatn. The highest *F_ST_* was 0.67 between PL- and SB charr, while the *F_ST_* was 0.03 on average for 10 microsatellites between these morphs [63]. This is in contrast to very little difference in *MHCIIα* variation among 9 Arctic charr populations from around Iceland (Figure 4). It is quite surprising to discover large differences at the *MHCIIα* among morphs within one lake, while the populations around Iceland were very similar. The pattern for *Cath2* was different. A modest *F_ST_* of 0.23 among morphs in Lake Thingvallavatn is notably (∼8X) higher than the *F_ST_* for microsatellites [63]. On a larger geographic scale, we observe very large *F_ST_* ‘s at *Cath2* among populations (highest 0.85). However there is no association of *Cath2* polymorphism with morphotype, while there may be a connection between relatedness and *Cath2* variation. The extent of differentiation in this locus is however stronger than seen in any individual microsatellite marker. In the absence of population genetic data spanning the relevant genomic regions, we cannot test for positive selection on those (or neighboring) genes.

Coalescence simulations [63] based on microsatellites (on the same fish studied here) support a model of very limited gene flow among the PL- and SB morphs in Lake Thingvallavatn, for the last 10.000 years. Also, the observed variation in microsatellites among arctic charr populations in Iceland and Lake Thingvallavatn, suggests substantial standing genetic variation in the anadromous stock(s) that colonized Icelandic waters. The reduced gene flow, due to isolation of populations or morphs, and local selective pressures could thus lead to differentiation in loci with fitness consequences. Thus the observed patterns in *MHCIIa* and *Cath2* within Lake Thingvallavatn and between Icelandic populations may reflect chance, history, and/or interplay of isolation and selection.

### Reduced Variation in the *MHCIIα* in Iceland?

One feature in the data demands special attention. MHC loci often exhibit extreme polymorphism and signs of balancing selection in fish systems [19]. In Iceland *MHCIIα* variation is very much reduced in all populations, except for the PL morph in Lake Thingvallavatn (which has two common haplotypes). Conejeros and colleagues [26] studied *MHCIIα* variation in 6 populations of Arctic charr across Europe, Asia and North America, and found much higher diversity (7 or more haplotypes in 5 populations; at most 14 individuals sequenced in each). Only the population from Trinité (2 haplotypes at 50% frequency in 9 individuals) had comparable level of variation to that observed in Lake Thingvallavatn PL charr. Part of the explanation may be that, we are studying a slightly shorter fragment of the *MHCIIα* locus than Conejeros and associates [26]. Many studies have documented excessive variation in MHC genes within and between fish populations, but there are also examples of local differences, in part attributable to natural selection [19].

The low diversity in *MHCIIα* among Icelandic Arctic charr populations may reflect history, for instance low diversity within the colonizing stock or a bottleneck in recent history. Alternatively strong selection for certain *MHCIIα* alleles in specific populations may also have played a role. A putative case in point is is the observation that the PL-charr is clearly distinct from the two benthic morphs in Lake Thingvallavatn. MHC driven mate choice has been extensively studied, with documented examples of both assortative and disassortative mating [86]–[88]. Eizaguirre and Lenz [19] conclude that under parasite mediated selection, MHC mediated assortative mate-choice could promote local adaptation and divergence. Our data cannot be used to evaluate such scenarios, but it would be interesting to test whether *MHCII* variation correlates with mating preferences of Arctic charr.

### *F_ST_* mapping and Putative Functional Alleles

*F_ST_* mapping can reveal both loci under positive selection and genes with relaxed purifying selection in certain populations, that stand out of the distribution of neutral variation. In this study a small fraction of the genome was interrogated and candidates were selected based on prior data and focus on particular pathways. This approach, although unlikely to find genes with the strongest signal of differentiation between groups, provided curious patterns for the sequenced candidates. In future genome wide single base polymorphism [89], microsatellite [90], [91], Rad-tag screens [92], [93] or even next generation sequencing of transcriptomes [94], [95] from distinct populations/species are interesting strategies to study this system in more detail.

The *MHCII* genomic regions have been cloned and sequenced in *S. salar* [96], but not in *S. alpinus*. In light of the results, it would be most interesting to clone and sequence the MHCII regions from Arctic charr, possibly from distinct morphs, populations or continents. Also, in salmon the regions contain several immunological genes, so differentiation at *MHCIIα* could be caused by linked variants in other genes [31]. As we studied only a part of intron 2 in *MHCIIα* it is rather unlikely that functional polymorphism(s) were surveyed in the data. The situation is different with *Cath2* where the strongest signal was a segregating polymorphism that leads to an amino acid replacement, serine to an arginine (S115R), in the predicted antimicrobial peptide region. Cathelicidins are like most AMPs cationic and target specifically the negative charged bacterial membrane, which ultimately leads to the killing of the bacteria [34]. It has been suggested that Cod cathelicidins (codCath) kill bacteria through lysis [44], but so far little is known about the functional mechanisms of other fish cathelicidins, which are less charged than codCath. Therefore it is difficult to speculate on the effect an amino acid change in the mature cathelicidin antimicrobial peptide in Arctic charr. Phylogenetic comparisons show that positive selection operates on charged amino acids in AMPs [45]. Thus it is tempting to speculate that the Cath2 S115R replacement is functional. One way to test whether *Cath2* is under positive selection is to assess *F_ST_* s along the locus and neighboring regions, to identify the marker with strongest signal of genetic differentiation between morphs and test formally for positive selection [97].

### Tests of Association of Genes and Ecological Attributes

Several studies in *S. salar* and related species reveal strong differentiation in immunological genes among populations or morphotypes [27], [98], [99], which may be in part due to differences in parasite diversity in distinct habitats. Eizaguirre et al. [100] demonstrated with an experimental set up that parasitic nematode infections change *MHCIIβ* allele frequencies in a single generation. Here we tested for association of four classes of large and prevalent parasites (*Diplostomum sp*., *Diphyllobothrium sp., Eubothrium salvelini* and Nematodes) and the *MHCIIα* haplotypes, but found no significant associations. This does not formally exclude the possibility that those parasites were not involved in shaping *MHCIIα* diversity, for methodological and other reasons. On the methodology side, the sample size is relatively small, compared to association tests in human genetics [101], [102] and the phenotypes are not measured in controlled environment as in quantitative genetics [103], [104]. Also, we only tested for association in a sample of 4–10 year old fish from 2010, but an association may have been between the genotype and parasites in the past (over many generations or during episodes of high infection) or only in juveniles. Reverse quantitative genetics can identify ecological variables of importance and shed light on the interplay of history, population genetic and ecological factors. However, failure of such phenotype hunts do not devalue the genetic signatures of differentiation among groups. QTL mapping within Arctic charr populations have identified chromosome regions that relate to ecologically important traits, *e.g.* spawning time and development [58], [105], [106].By combining population genetic and QTL mapping techniques, loci related to adaptation can be identified [107].

### Freshwater Fishes to Study Adaptation

Following the last glaciation Nordic freshwater fishes expanded into new territories. Several features, like novel habitats, geographic isolation of stocks, in some cases small population sizes or bottlenecks, reduced gene flow and the relatively simpler ecosystem of arctic areas, could lead to rapid evolution via both drift and selection. Some Arctic charr populations show dedicated resource morphotypes while others retain ancestral phenotypes [8], [108]. Similar to the stickleback and Mexican cavefish [9], [109] the dozens of morphologically and ecologically distinct Arctic charr populations are *de facto* natural experiments in parallel evolution [53], [63]. Genome-wide markers make it possible to elucidate the history of the distinct and even sympatric populations [93], [107], [110] and identify genes relating to adaptation [14], [15], [95], [111]. Northern species like Arctic charr, which have invaded similar habitats multiple times and adapted to them in relatively short evolutionary time, provide an interesting system to dissect the genetics and ecology of parallel evolution, however complicated and challenging.

## Supporting Information

Table S1 Specifics of primers and annealing temperatures.
(DOC)

Table S2 MHCIIα genotyping and polymorphism.
(DOC)
