# Supplementary material for: Differentiation at the MHCIIα and Cath2 Loci in Sympatric Salvelinus alpinus Resource Morphs in Lake Thingvallavatn
Source: PLoS One. 2013 Jul 24;8(7):e69402. doi: 10.1371/journal.pone.0069402 (PMC3722248; doi:10.1371/journal.pone.0069402)
Supplement: Table S1 — Specifics of primers and annealing temperatures. (DOC) [file pone.0069402.s001.doc]

Supplemental table S1 for **Differentiation at the *MHCIIα* and *Cath2* loci in sympatric *Salvelinus alpinus* resource morphs in Lake Thingvallavatn** Kalina H. Kapralova, Johannes Gudbrandsson, Sigrun Reynisdottir, Cristina B. Santos, Vanessa C. Baltanás, Valerie H. Maier, Sigurdur S. Snorrason and Arnar Palsson.

Supplemental Table S1. Specifics of primers and annealing temperatures.

| Locus | Primer name | Primer sequence 5'-3' | Ta (°C) |
| --- | --- | --- | --- |
| *MHCIIα* | *SAALDAAF** | CTGGATGCAGTGATTCAGATG | 53 |
|  | *SAALDAAR** | GACGTGGCAGATGAGAGTG |  |
|  | SAMHC2a_f8 | CAAGAACCCACCAGAGACAA | 53 |
|  | SAMHC2a_r5 | TGGGAACACATTTAGCATCA |  |
| D-loop | SADloop_F | CCACCCTTAACTCCCAAAGC | 57 |
|  | SADloop_R | GGCTTGGTGGGTAACGAAC |  |
| *Hepcidin* | SAHep_F | TACGCTGGCCCTTTTCTACA | 53 |
|  | SAHep_R | CTTTCTCCCTGGGTGCATTA |  |
| *Leap-2a* | SALeap2a_F | GATATTGAATGCTAGCTTTTGGAC | 53 |
|  | SALeap2a_R | AAAGGCCATTGCAAAGACAG |  |
| *Cath2* (3' UTR) | SACat_F5 | AGCAAGGCCAACCATGTC | 57 |
|  | SACat_R5 | TGCAGTAAACATGAACTGGAAA |  |
| *Cath2* (peptide) | SACat_f9 | GGAGACGCTCTGCAGTAAGG | 57 |
|  | SACat_r9 | GGTTTACTCCGCTAGCTCCA |  |
| *Cath2* (intron 2) | SACat_f7 | AAATCAGCTGCTTCCTGTGG | 57 |
|  | SACat_r8 | GAGGACATGGTTGGCCTTG |  |

* From Conejeros et al 2008.
