# Supplementary material for: Differentiation at the MHCIIα and Cath2 Loci in Sympatric Salvelinus alpinus Resource Morphs in Lake Thingvallavatn
Source: PLoS One. 2013 Jul 24;8(7):e69402. doi: 10.1371/journal.pone.0069402 (PMC3722248; doi:10.1371/journal.pone.0069402)
Supplement: Table S2 — MHCIIα genotyping and polymorphism. (DOC) [file pone.0069402.s002.doc]

Supplemental table S2 for **Differentiation at the *MHCIIα* and *Cath2* loci in sympatric *Salvelinus alpinus* resource morphs in Lake Thingvallavatn** Kalina H. Kapralova, Johannes Gudbrandsson, Sigrun Reynisdottir, Cristina B. Santos, Vanessa C. Baltanás, Valerie H. Maier, Sigurdur S. Snorrason and Arnar Palsson.

Supplemental Table S2. *MHCIIα* genotyping and polymorphism.

| Genotyping | Reference | Haplotype | S186 | S236 | S269 | S271 | I280 | S294 | S346 | S349 | S362 | S375 | S458 | S472 |
| --- | --- | --- | --- | --- | --- | --- | --- | --- | --- | --- | --- | --- | --- | --- |
| Cloned | Saal-DAA*0801 (99%) |  | A | T | C | A | 1 | G | A | A | G | A | A | C |
| Cloned and PCR | Saal-DAA*0305/0306/0307 (98%) | hap14 | C | G | A | T | 1 | G | A | T | A | A | A | T |
| Cloned and PCR | Saal-DAA*0305 (99%) | hap15 | C | G | A | T | 0 | A | G | T | G | G | T | T |

Positions in reference to EF450451.1 (Saal-DAA*0305)

Reference indicates the best hit in Genebank (% identity from Blastn)

Cloned= Cloned and sequenced

PCR = direct PCR and sequencing
